# Chronic Endometritis in Infertile Women: Impact of Untreated Disease, Plasma Cell Count and Antibiotic Therapy on IVF Outcome—A Systematic Review and Meta-Analysis

**DOI:** 10.3390/diagnostics12092250

**Published:** 2022-09-18

**Authors:** Amerigo Vitagliano, Antonio Simone Laganà, Dominique De Ziegler, Rossana Cicinelli, Carla Mariaflavia Santarsiero, Giovanni Buzzaccarini, Vito Chiantera, Ettore Cicinelli, Marco Marinaccio

**Affiliations:** 1 Unit of Obstetrics and Gynecology, Department of Biomedical and Human Oncologic Science, University of Bari, 70100 Bari, Italy; 2 Unit of Gynecologic Oncology, ARNAS “Civico-Di Cristina-Benfratelli”, Department of Health Promotion, Mother and Child Care, Internal Medicine and Medical Specialties (PROMISE), University of Palermo, 90127 Palermo, Italy; 3 Department of Obstetrics, Gynecology, and Reproductive Medicine, Hospital Foch, Faculté de Médecine Paris Ouest (UVSQ), 92150 Suresnes, France; 4 Unit of Gynecology and Obstetrics, Department of Women and Children’s Health, University of Padua, 35128 Padua, Italy

**Keywords:** chronic endometritis, endometritis severity, infertility, IVF outcome, antibiotic therapy, plasma cell count, CD-138 immunohistochemistry, hysteroscopy, recurrent implantation failure

## Abstract

This systematic review and meta-analysis aims to evaluate the impact of chronic endometritis (CE) and its therapy on in vitro fertilization (IVF) outcome. Additionally, we aim to investigate whether various degrees of CE severity may exert a different effect on IVF outcome. Ongoing-pregnancy rate/live-birth-rate (OPR/LBR), clinical-pregnancy rate (CPR), and miscarriage rate (MR) were calculated. A total number of 4145 patients (from ten studies) were included. Women with CE had lower OPR/LBR (OR 1.97, *p* = 0.02) and CPR (OR 2.28, *p* = 0.002) compared to those without CE. CE cure increased OPR/LBR (OR 5.33, *p* < 0.0001) and CPR (OR 3.64, *p* = 0.0001). IVF outcome was comparable between women with cured CE and those without CE (OPR/LBR, CPR and MR: *p* = ns). Women with severe CE had lower OPR/LBR (OR 0.43, *p* = 0.003) and CPR (OR 0.40, *p* = 0.0007) compared to those mild CE. Mild CE showed no influence on the IVF outcome as compared to women without CE (OPR/LBR, CPR and MR: *p* = ns). Based on this data analysis, CE significantly reduces OPR/LBR and CPR in women undergoing IVF. Importantly, CE resolution after antibiotic therapy may improves IVF outcome, leading to similar OPR/LBR and CPR as compared to unaffected patients. The negative effects of CE on IVF outcome may be restricted to severe disease, whereas mild CE may have no influence on IVF success.

## 1. Introduction

Chronic endometritis (CE) is a chronic inflammatory state of the endometrium caused by an abnormal endometrial microbiome [1]. In recent years, a growing interest in CE has been seen, especially due to its putative role in infertility, recurrent pregnancy loss, and repeated IVF failures (RIF) [2,3,4,5,6,7]. Notably, in these conditions, CE prevalence has been often reported to exceed 30% [8,9,10].

Different theories have been proposed for explaining CE-related impaired endometrial receptivity [11,12,13,14], including the activation of local inflammatory processes with altered cytokine and chemokine secretion [13,15,16,17,18], abnormal leukocyte infiltration within the endometrium [19,20], altered uterine contractility [21], defective decidualization [17,22], and defective endometrial vascularization [13,23].

Although these theories are certainly intriguing, available evidence regarding correlation between CE and implantation defects is mainly based on observational data from studies with some shortcomings (e.g., heterogeneous design and criteria for diagnosing CE) [24]. Therefore, the scientific community remains divided between researchers who are for and those who are against recognizing that CE is a real cause of female infertility.

One of the crucial issues regarding CE is the methodology used for its diagnosis. Hysteroscopy has a fair sensitivity but suffers from being strongly operator dependent [24]. For this reason, the current gold standard for CE diagnosis is syndecan-1 staining of plasma cells on endometrial tissue sections, alone or in combination with multiple-myeloma antigen 1 immunohistochemistry [25,26]. Yet, the amount of plasma cells per sample/area or microscope field for diagnosing CE remains controversial [27].

In a previous systematic review with meta-analysis of data from five studies [28], we found that CE therapy with antibiotics could improve the IVF outcome in women with a history of repeated IVF failure. In this present review, we extended our earlier evaluation to all studies on infertile women undergoing IVF. Specifically, we tested whether CE may worsen IVF outcome. Additionally, we evaluated the effects of CE cure on IVF outcome. Finally, we also investigated whether various degrees of CE severity (i.e., entity of plasma cell infiltration) may exert a different effect on the IVF outcome.

## 2. Materials and Methods

### 2.1. Study Design

This is a systematic review and meta-analysis of published data (PROSPERO ID: CRD42017062494). The review was reported following the Preferred Reporting Items for Systematic Reviews and Meta-Analyses (PRISMA) guidelines [29].

### 2.2. Search Strategy

Electronic databases (Sciencedirect, Medline, Scopus, Embase, the Cochrane library, Clinicaltrials.gov, EU Clinical Trials Register, and the World Health Organization International Clinical Trials Registry) were searched from their inception through 31 December 2021.

Key search terms were: chronic endometritis OR endometrial inflammation OR endometrial plasma cells OR endometrial CD-138 count AND IVF OR ICSI OR embryo transfer OR ARTs. The electronic search and the eligibility of the studies were independently assessed by two authors (A.V., E.C.).

### 2.3. Inclusion Criteria

We included all studies evaluating the effects of CE on IVF-ET outcome in infertile patients. Studies on women with a history of recurrent miscarriage were excluded. Additionally, all studies evaluating the rates of spontaneous conception in women with CE were not eligible for inclusion.

All original studies (experimental and observational) reported in the English language were evaluated. CE was defined as the presence of at least one endometrial stromal plasma cell in the entire section, as demonstrated by immunohistochemistry for CD-138 (syndecan-1). “Severe CE” was defined as the presence of ≥5 plasma cells/HPF. “Mild CE” was defined as the presence of 1–4 plasma cells/HPF within endometrial tissue.

All studies evaluating other types of endometrial inflammation (such as acute, subacute, or tubercular endometritis) were excluded.

### 2.4. Comparators

- Patients with CE vs. non-CE: defined as patients suffering from CE (i.e., untreated or persistent after antibiotic therapy) versus those without CE (with normal endometrial histology);
- Patients with CE vs. cured CE: defined as patients suffering from CE (i.e., untreated or persistent after antibiotic therapy) versus those in which (after antibiotic therapy) endometrial biopsy showed the resolution of CE;
- Patients with cured CE vs. non-CE: defined as women with CE resolution (after antibiotic therapy) versus women without CE (with normal endometrial histology);
- Patients with CE vs. not tested for CE: defined as patients with CE (i.e., untreated or persistent after antibiotic therapy) versus those in which CE was not investigated.

Secondary analyses included the comparison between patients with CE as defined by the presence of ≥5 plasma cells/HPF (“severe CE”) vs. 1–4 plasma cells/HPF (“mild CE”) within endometrial tissue. Additionally, the subgroup of patients with 1–4 plasma cells/HPF was compared with patients without CE.

### 2.5. Study Outcomes

Study outcomes included ongoing pregnancy or live birth rate (per patient [OPR/LBR]), clinical pregnancy rate (per patient [CPR]), miscarriage rate (per clinical pregnancy [MR]).

Outcomes measures:

- OPR/LBR: “Ongoing pregnancy” defined as a pregnancy beyond 12 weeks’ gestation; “live birth” defined as the delivery of one or more living infants;
- CPR: defined as the presence of a gestational sac on transvaginal ultrasound or other definitive clinical signs;
- MR: defined as fetal loss prior to the 20th week of gestation.

### 2.6. Study Selection and Data Extraction

Three authors (A.V., C.M.S., R.C.) independently performed the study selection. Disagreements were discussed with a third reviewer (E.C.).

Data extraction was performed independently by two authors (A.V., C.M.S.). A manual search of the reference list of each study was performed to avoid missing relevant publications. One Author (E.C.) completely reviewed the selection and data extraction process. Results were compared, and any disagreement was resolved by consensus.

### 2.7. Risk of Bias in Individual Studies

Two reviewers (A.V., C.M.S.) independently judged the methodological quality of the studies included in the meta-analysis using a modified version of the “Newcastle–Ottawa Scale” [30]. The quality of the studies was evaluated in five different domains: “sample representativeness”, “sampling technique”, “ascertainment of chronic endometritis diagnosis”, “quality of description of the population”, and “incomplete outcome data” (Appendix A). According to the total number of points assigned, each study was judged to be at low risk of bias (≥3 points) or high risk of bias (<3 points). Any discrepancies concerning the authors’ judgements were referred to a third reviewer (E.C.) and resolved by consensus.

### 2.8. Statistical Analysis

Data analysis was performed independently by two authors (A.V., E.C.) with Review Manager version 5.3 (Nordic Cochrane Centre, Cochrane Collaboration, Copenhagen, Denmark). The study outcomes were expressed using odds ratio (OR) with 95% confidence interval (95% CI); *p* values lower than 0.05 were considered statistically significant. The I^2^ statistics was used to assess heterogeneity. The degree of heterogeneity was considered as low when I^2^ was <30%, moderate if it was between 30% and 50%, and high if I^2^ was >50%. Random-effects model (DerSimonian and Laird method) was applied to the meta-analyses. Subgroup and sensitivity analyses were also planned in order to explore the sources of heterogeneity across studies (when at least four studies were included in the meta-analysis). We followed Cochrane Handbook recommendations for the assessment of publication bias (Cochrane Handbook. 10.4.3.1 Recommendations on testing for funnel plot asymmetry) [31]. However, not enough studies (fewer than ten) were included in the pooled analysis.

## 3. Results

### 3.1. Study Selection

**Figure 1:**
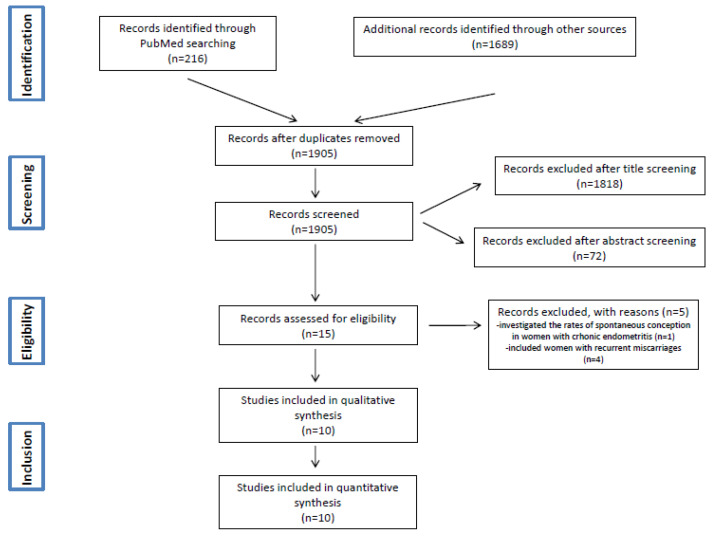
PRISMA flow-chart of study screening, selection, and inclusion/exclusion.

After the evaluation of the full text, a total number of ten studies [4,6,8,9,10,11,12,32,33,34] were included in the present meta-analysis (Figure 1).

### 3.2. Included Studies

Studies included a total of 4145 patients. All studies were observational: four prospective studies [6,12,32,34], five retrospective studies [4,9,10,11,33], and one cross-sectional study [8].

Two studies compared non-CE patients, patients with cured CE, and patients with persistent CE [12,34]. One study compared patients with cured CE and patients with persistent CE [4]. Two studies compared non-CE patients, patients with cured CE, and patients not tested for CE [6,11]. Two studies compared patients with CE and patients without CE [32,33]. One study compared patients with CE, patients without CE, and patients with cured CE [8]. Li et al. [9] divided their patients into six groups based on the number of CD138^+^ cells per HPF (0/HPF, 1/HPF, 2/HPF, 3/HPF, 4/HPF, and ≥5/HPF) and compared pregnancy outcome in women with <5/HPF and ≥5/HPF plasma cells.

Xiong et al. [10] studied different subgroups of patients as well, based on the number of CD138^+^ cells (0/HPF, 1–4/HPF, ≥5/HPF) and compared pregnancy outcome between women with CD138^+^/HPF < 5 and women with persistent CE after antibiotic therapy. The characteristics of all included studies are summarized in Appendix B.

### 3.3. Patients

Four studies included patients with RIF [4,6,11,12,34]. RIF was defined as the failure of at least two or three previous (fresh or frozen-thawed) IVF-ET attempts, including at least one good-quality cleavage-stage embryo or blastocyst transferred per cycle. One study included patients who had experienced only one previous embryo transfer failure [33]. Four studies analyzed infertile patients with unselected previous ET [8,9,10,32].

### 3.4. IVF-Embryo Transfer Cycle

All patients underwent IVF. Information about IVF-ET protocols were not available for three studies [9,12,33], whereas seven studies reported adequate information about IVF-ET protocols. Ovarian stimulation was performed through the daily administration of recombinant FSH (rFSH) alone or in combination with human menopausal gonadotropin (hMG), using GnRH-ant (fixed or flexible protocol) or GnRH-a (long protocol) for pituitary desensitization. U-hCG (5000–10,000 IU) was administered when at least two pre-ovulatory (17 mm) follicles were identified on a transvaginal ultrasound scan. Egg retrieval was performed 34–36 h after ovulation induction and no more than three embryos or two blastocysts per cycle were transferred. Specifically, in two studies [4,34] only cleavage-stage embryos (up to three) were transferred, whereas in the study by Hirata and Kuroda [8,32], only blastocysts were transferred. In two studies [10,11], embryo transfers were performed at either the cleavage or blastocyst stage. No data were available on embryo stage in the study reported by Johnston-MacAnanny and coworkers [6]. Luteal phase support with either vaginal or intramuscular progesterone was administered in all the studies that reported information about their protocols.

### 3.5. Diagnosis of Chronic Endometritis

Plasma cells identification was achieved with hematoxylin and eosin (H&E) staining alone or in combination with immunohistochemical (IHC) examination for CD-138, except in Fan et al. [33], who preferred to use only immunohistochemical (IHC) examination for CD-138. Endometrial specimens were collected during the follicular phase in six studies [4,10,12,32,33,34]; Demirdag et al. [11] performed endometrial biopsy either in the follicular phase of the cycle or mid-luteal phase (cycle days 21–23). In two studies, endometrial biopsy was performed in mid-luteal phase [8,9]. No information was obtained in the report made by Johnston-MacAnanny et al. [6].

The diagnosis of CE was made by a single, expert pathologist in four studies [4,6,12,32]. In the studies of Fan et al. [33] and Li et al. [9], two experienced pathologists independently performed the identification and counting of CD138^+^ cells. The diagnosis of CE was made by different experienced pathologists in three studies [8,10,34]. Demirdag et al. [11] did not report any information about the number of the pathologists who evaluated the biopsies.

### 3.6. Therapy of Chronic Endometritis

First line antibiotic therapy for CE was germ-specific when endometrial culture was performed [4,8,34] or empiric: doxycycline 200 mg/day for 14 days [6,10,12] or ciprofloxacin 1 g/day and metronidazole 1 g/day for 14 days [11].

### 3.7. Assessment of Study Quality and Risk of Bias

- Sample representativeness: three studies had adequate sample representativeness [4,11,33]. Remaining studies were judged at a high risk of bias [6,8,9,10,12,32,34].
- Sampling technique: three studies had adequate sampling strategy (consecutive) [8,11,12]. The majority of studies did not provide precise information [4,6,9,10,32,33,34].
- Ascertainment of chronic endometritis diagnosis: all studies were at low risk of bias [4,6,8,9,10,11,12,32,33,34].
- Quality of population description: two studies failed to provide a clear description of the study population or incompletely reported descriptive statistics [12,34]. Remaining studies were at low risk of bias for this domain [4,6,8,9,10,11,32,33].

Incomplete outcome data: Three studies provided incomplete outcome data [11,12,33].

According to the total number of points assigned, all studies were judged at low risk of bias (≥3 points) [4,6,8,9,10,11,12,32,33,34] (Table 1).

Assessment of publication bias was not possible because not enough studies (fewer than ten) were included in pooled analysis for the primary outcome.

### 3.8. Synthesis of Results

- CE vs. non-CE

**Figure 2:**
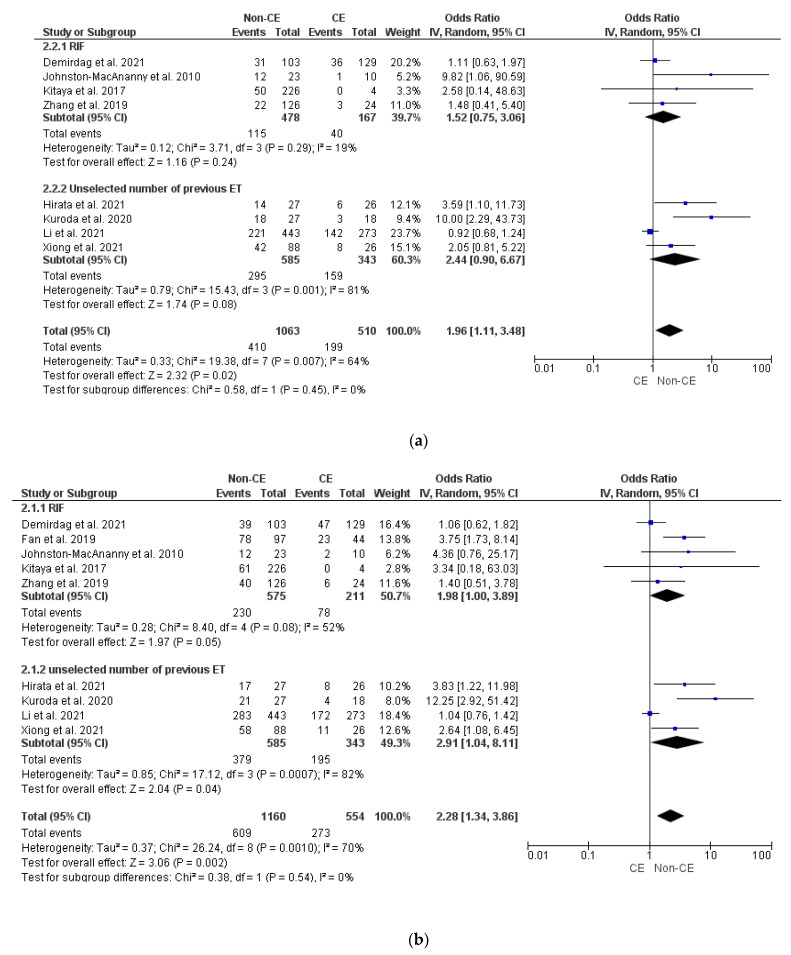

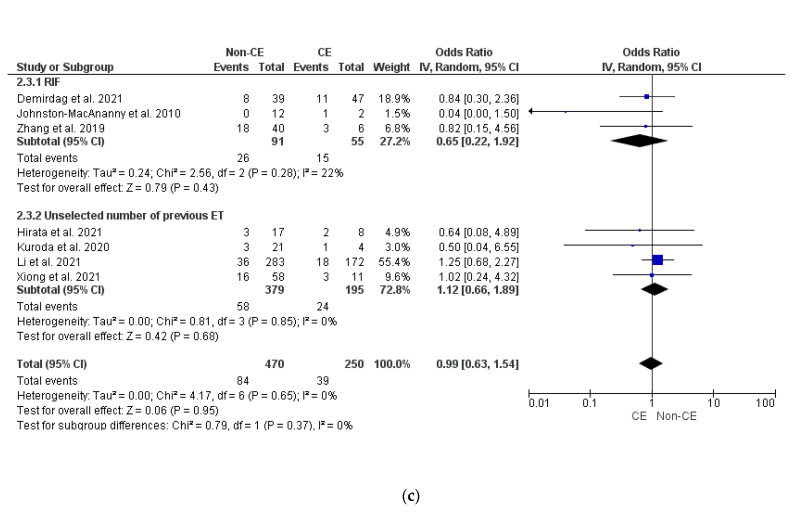
Pooled data analysis comparing CE versus non-CE: (**a**) ongoing pregnancy rate/live birth rate; (**b**) clinical pregnancy rate; (**c**) miscarriage rate [6,8,9,10,11,12,32,33,34].

Data from eight studies [6,8,9,10,11,12,32,34] showed significantly lower OPR/LBR (OR 1.97, 95% CI 1.11–3.48, I^2^ = 64%, *p* = 0.02) and CPR (OR 2.28, 95% CI 1.34–3.86, I^2^ = 70%, *p* = 0.002) in patients with CE in comparison to those without CE, with no difference in terms of MR (*p* = ns) (Figure 2a–c). The serial exclusion of each study from meta-analysis did not provide substantial changes to pooled results in terms of OPR/LBR, CPR, and MR. Subgroup analysis based on the number of previously failed ET did not find statistical differences (*p* = ns) (Figure 2a–c).

- CE vs. cured CE

**Figure 3:**
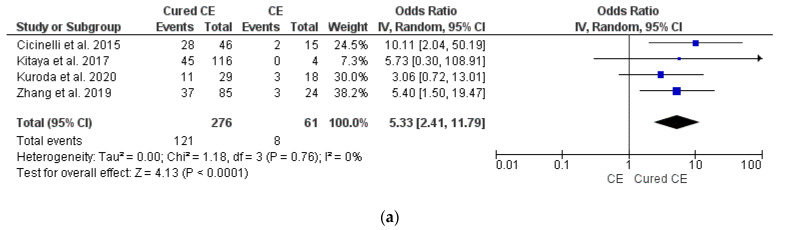

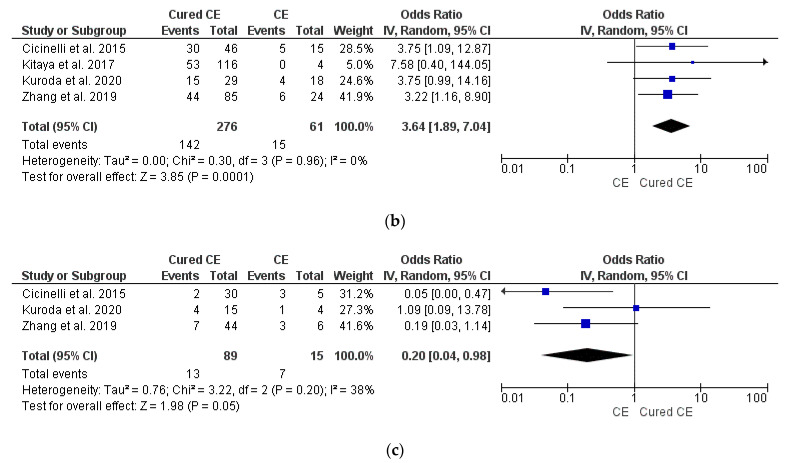
Pooled data analysis comparing CE versus cured CE: (**a**) ongoing pregnancy rate/live birth rate; (**b**) clinical pregnancy rate; (**c**) miscarriage rate [4,8,12,34].

We found higher OPR/LBR (OR 5.33, 95% CI 2.41–11.79, I^2^ = 0%, *p* < 0.0001) and CPR (OR 3.64, 95% CI 1.89–7.04, I^2^ = 0%, *p* = 0.0001) in patients with cured CE in comparison to those with untreated/persistent CE (data from four studies [4,8,12,34]), with borderline significance in terms of MR (*p* = 0.05) (Figure 3a–c). The serial exclusion of single studies from meta-analysis did not provide substantial changes to pooled results for OPR/LBR and CPR. Sensitivity analysis was not feasible for MR. Subgroup analysis based on the number of previously failed ET did not find statistical differences (*p* = ns; data not shown).

- Cured CE vs. non-CE

**Figure 4:**
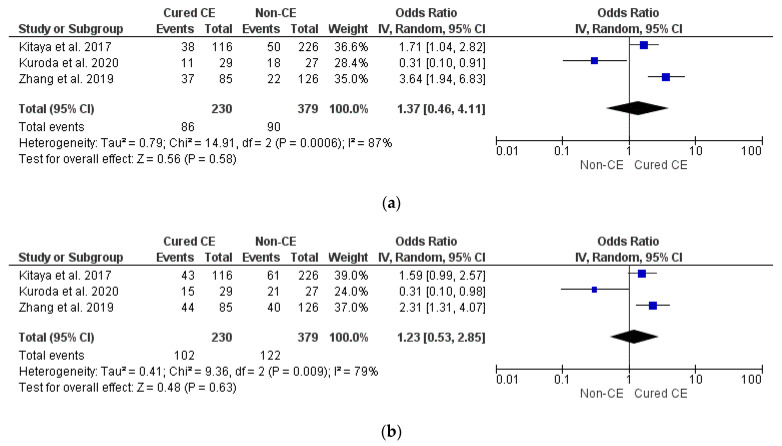

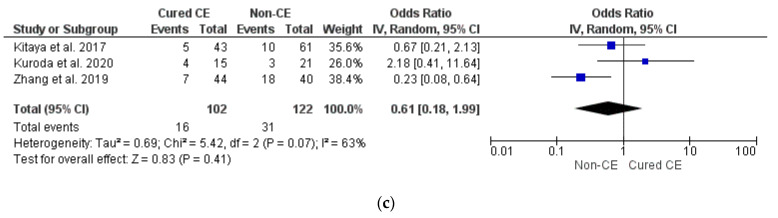
Pooled data analysis comparing non-CE versus cured CE: (**a**) ongoing pregnancy rate/live birth rate; (**b**) clinical pregnancy rate; (**c**) miscarriage rate [8,12,34].

Analysis of 609 patients from three studies [4,8,34] did not show any difference between groups in terms of OPR/LBR, CPR, and MR (*p* = ns) (Figure 4a–c). Sensitivity and subgroup analyses were not feasible (*n* = 3 studies included in meta-analysis).

- CE vs. non-tested for CE

**Figure 5:**
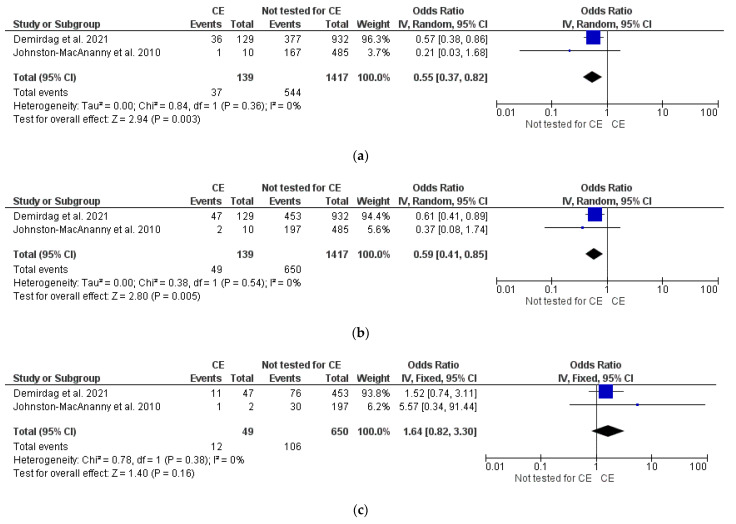
Pooled data analysis comparing CE versus non-tested for CE: (**a**) ongoing pregnancy rate/live birth rate; (**b**) clinical pregnancy rate; (**c**) miscarriage rate [6,11].

Pooled analysis of data on 1556 patients from two studies [6,11] showed lower OPR/LBR (OR 0.55, 95% CI 0.37–0.82, I^2^ = 0%, *p* = 0.003) and CPR (OR 0.59, 95% CI 0.41–0.85, I^2^ = 0%, *p* = 0.005) in women with untreated CE compared to those not tested for CE. No difference was found in MR (*p* = ns) between comparators (Figure 5a–c). Sensitivity and subgroup analyses were not feasible (*n* = 2 studies included in meta-analysis).

- Severe CE vs. mild CE

**Figure 6:**
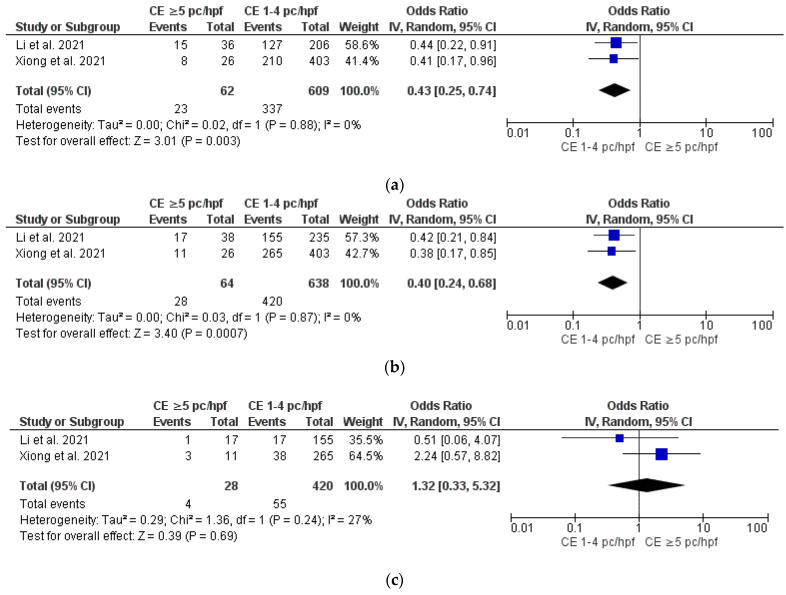
Pooled data analysis comparing severe versus mild CE: (**a**) ongoing pregnancy rate/live birth rate; (**b**) clinical pregnancy rate; (**c**) miscarriage rate [9,10].

Data from two studies [9,10] showed that severe CE (≥ 5 plasma cells/HPF) was associated with significantly lower OPR/LBR (OR 0.43, 95% CI 0.25–0.74, I^2^ = 0%, *p* = 0.003) and CPR (OR 0.40, 95% CI 0.24–0.68, I^2^ = 0%, *p* = 0.0007) compared to mild CE (1–4 plasma cells/HPF), with no difference in MR (Figure 6a–c). Sensitivity and subgroup analyses were not feasible (*n* = 2 studies included in meta-analysis).

- Mild CE vs. non-CE

**Figure 7:**
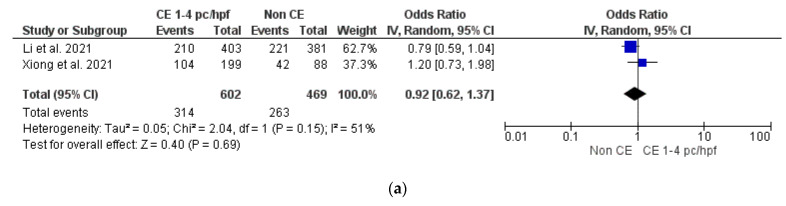

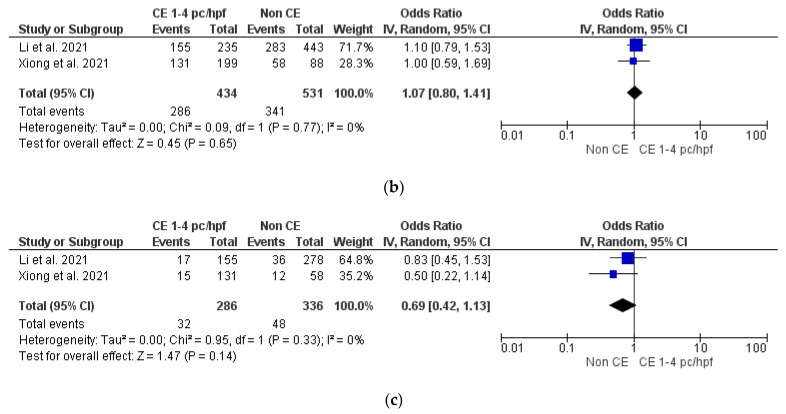
Pooled data analysis comparing mild CE versus non-CE: (**a**) ongoing pregnancy rate/live birth rate; (**b**) clinical pregnancy rate; (**c**) miscarriage rate [9,10].

No difference was found between groups [9,10] in terms of OPR/LBR, CPR and MR (*p* = ns). Sensitivity and subgroup analyses were not feasible (*n* = 2 studies included in meta-analysis) (Figure 7a–c).

## 4. Discussion

### 4.1. Main Findings and Implications

This present systematic review summarized for the first time the available evidence on the impact of CE, its cure, and severity on IVF outcome. The analysis included a total of 4145 infertile patients from 10 observational studies [4,6,8,9,10,11,12,32,33,34], of which 1716 were women with RIF. The overall quality of the included studies was fair (no study judged at high risk of bias).

Importantly, women without CE showed significantly higher OPR/LBR (OR 1.97, 95% CI 1.11–3.48) and CPR (OR 2.28, 95% CI 1.34–3.86) compared to women suffering from CE. This finding, consistent across all study populations with variable numbers of previously failed embryo transfers (*p* < 0.05), reinforces our previous results on the negative effects of CE on embryo implantation [28] and extends the evidence to all women undergoing IVF. In addition, the analysis is now more robust, as it relies on a large number of studies [4,6,8,9,10,11,12,32,33,34] with unambiguous diagnostic approaches for the diagnosis of CE (i.e., CD-138 immunohistochemistry).

Several factors may be involved in CE-related impaired reproductive failure [14]. The disease is primarily caused by abnormal intrauterine bacterial proliferation, as demonstrated by microbiological studies [1,35,36], and further confirmed by a high rate of CE cure after antibiotic therapy [26,37]. Intrauterine infection leads to a specific cytokine and leukocyte pattern in order to prepare the uterus to fight the noxa [13,16]. Specifically, the immunosuppression needed for embryo implantation is converted into an immunoreaction. On the one hand, such a reaction may disrupt the embryo–endometrial crosstalk and hamper the process of blastocyst invasion [14]. On the other hand, sustained up-regulation of proliferative genes and down-regulation of apoptotic genes [18] (required for endometrial reaction) may promote the development of proliferative lesions such as (micro and macro) polyps [18,38,39]. Moreover, significant and severe alterations in the vascularization and decidualization of secretory endometrium [6,40] may further contribute to receptivity impairment in CE.

Interestingly, women with CE showed also poorer IVF outcomes compared to a group of patients not screened for CE (OPR/LBR: OR 0.55, 95% CI 0.37–0.82; CPR: OR 0.59, 95% CI 0.41–0.85; *p* < 0.05). This comparison was based on data from two studies [6,11], and our confidence in the effect estimate is therefore limited. It implies that the data should be interpreted with caution. Nevertheless, if this result is confirmed by future studies, it may theoretically justify offering CE screening before IVF for the identification (and treatment) of a subgroup of women with expected poor reproductive prognosis. This principle is reinforced by the significant improvement of the IVF outcome after CE cure emerging from additional analyses included in this review. Indeed, the OPR/LBR and CPR after CE cure were considerably higher compared to those of women with untreated or persistent CE (OR 5.33, 95% CI 2.41–11.79; OR 3.64, 95% CI 1.89–7.04; all *p* < 0.05), with low statistical inconsistency (I^2^ = 0%). Surprisingly, women with cured CE had similar IVF success compared to women without CE (*p* > 0.05), potentially suggesting a “restitutio ad integrum” of endometrial receptivity towards the embryo after the removal of CE.

Separate considerations are needed to simplify the interpretation of our findings on MR, which apparently deviate from those of other outcomes (OPR/LBR, CPR). Our meta-analysis found no effect of CE on MR, nor any advantages in terms of MR improvement after CE cure (borderline significance; *p* = 0.05). We can speculate that miscarriage has many different etiopathogenetic factors related to either the mother or the embryo, and therefore it can be only moderately influenced by CE (and its cure) [41,42,43,44]. In particular, embryo aneuploidy is considered as the principal factor of miscarriage, and advanced maternal age (≥35 years old) is the main risk factor [41,45,46]. In this regard, the majority of included studies enrolled also patients of advanced age up to 44 [9], 40 [32], 39 [4,10,11], and 38 [33] years old. Other studies (in which patients’ age cut-off was not specified) reported mean patients’ age close to 35 years old [6] or higher than 35 years old [8,12] and therefore included a certain proportion of women aged ≥35 years. No study applied pre-implantation genetic testing for aneuploidies. The only study on young women (<35 years old) was by Zhang et al. [34], in which women suffering from CE showed a trend towards higher MR compared to healthy women or women with cured CE. Another reflection about MR should account for statistical issues inherent to small sample sizes of the comparators, with high risk of type II error. Notably, any comparisons involving MR will be underpowered compared to those involving CPR or OPR/LBR in a definite study population. For instance, if we consider a number of patients (N) enrolled in a certain study, of whom 50% achieve a clinical pregnancy, the sample size for MR will be 50% lower than CPR and OPR/LBR (i.e., N/2). Accordingly, the total number of patients and events included in our review is insufficient to draw a definitive conclusion on the impact of CE and its cure on MR after IVF.

Non-conclusive but intriguing findings of our review come from our secondary analyses on the impact of severe CE (i.e., ≥5 plasma cells/HPF) and mild CE (i.e., 1–4 plasma cells/HPF) on the IVF outcome. Data from two studies showed that severe CE was associated with lower OPR/LBR (OR 0.43, 95% CI 0.25–0.74) and CPR (OR 0.40, 95% CI 0.24–0.68) than mild CE (*p* < 0.05). Nevertheless, women with mild CE showed similar OPR/LBR and CPR as compared to women without CE (*p* > 0.05). These data were consistent with the findings of Fan et al. [33] and Li et al. [9] in that the higher the number of cells expressing CD138, the worse the outcome of IVF. Although the opportunity to classify CE in mild and severe forms is tempting for practical reasons, available evidence is insufficient to consider “mild CE” (defined as 1–4 plasma cells/HPF) as a benign condition [27]. The choice to classify CE exclusively based on plasma cell counts is practical but potentially misleading. When endometrial biopsy is performed with a blind method (i.e., by using a Pipelle or a curette), the reliability of the CE classification may depend on the amount of endometrial tissue captured, especially if the distribution of plasma cells is heterogeneous throughout the endometrial surface. Moreover, in the case of focal CE, the disease can be underestimated due to the random nature of tissue collection, potentially sampling healthy areas of the uterine cavity. Last but not least, if plasma cells are counted based exclusively on CD138 staining, overestimation of CE may sometimes occur due to background reaction [25].

According to our recent experience, hysteroscopy may represent an add-on technique for the diagnosis of CE [5,24,26], especially in the case of diagnostic uncertainties [47,48]. Through a visual evaluation of the whole endometrial surface, hysteroscopy may allow the recognition of specific endometrial changes consistent with severe CE (e.g., micropolyps) [49]. In this regard, inconsistency has been previously demonstrated between the diagnoses of CE achieved by plasma cell count and those obtained by hysteroscopy [5,16,24]. For this reason, it cannot be excluded that the combination of the two techniques may provide higher diagnostic and prognostic value compared to immunohistochemistry alone. For example, in the study by Yang et al. [50], those patients in whom control hysteroscopy showed disappearance of CE “signs” had greater IVF success compared to women in whom immunohistochemistry demonstrated CE cure (i.e., no residual plasma cells). Last but not least, hysteroscopy may be a useful instrument for undertaking endometrial tissue sampling under visual control (e.g., sampling the areas in which CE is suspected) [51,52]. Nevertheless, although the use of hysteroscopy with direct biopsies is consolidated in the approach to focal endometrial lesions [53,54,55], its effectiveness has still not been evaluated for CE.

### 4.2. Strengths and Limitations

To the best of our knowledge, this is the largest meta-analysis evaluating the effects of untreated CE, its severity, and CE therapy on IVF outcome. Originality, rigorous methodology, and the inclusion studies with low risk of bias are the main strengths. Limitations are inherent to the small number of patients and studies included in specific analyses, heterogeneity in patients’ characteristics (including IVF cycles and days for embryo transfer [cleavage stage vs. blastocyst stage embryos]), variability in therapeutic schemes for CE among studies, and inclusion of patients of advanced age (≥35 years old) without adjusting for embryo aneuploidy.

## 5. Conclusions

CE may significantly reduce OPR/LBR and CPR in women undergoing IVF. Importantly, CE resolution after antibiotic therapy seems to improve the reproductive outcome in those women, leading to similar IVF outcomes as compared to unaffected patients.

Low quality evidence suggests that the negative effects of CE on IVF outcome may be restricted to severe disease (≥5 plasma cells/HPF), whereas mild CE (1–4 plasma cells/HPF) may be non-harmful for embryo implantation.

Future randomized controlled studies are needed to test the effectiveness of offering CE screening to the general IVF population with the purpose of improving OPR/LBR. Additionally, further studies assessing the impact of mild CE on IVF outcome and the usefulness of hysteroscopy in this condition are recommended.

## Figures and Tables

**Table 1:** Risk of bias assessment.

Authors and Year	Sample Representativeness	Sampling Technique	Ascertainment of CE Diagnosis	Quality of Description of the Population	Incomplete Outcome Data	Total Score	Risk of Bias
Cicinelli et al. 2015 [4]	★	-	★	★	★	★★★★	Low
Demirdag et al. 2021 [11]	★	★	★	★	-	★★★★	Low
Fan et al. 2019 [33]	★	-	★	★	-	★★★	Low
Hirata et al. 2021 [32]	-	-	★	★	★	★★★	Low
Johnston-MacAnanny et al. 2010 [6]	-	-	★	★	★	★★★	Low
Kitaya et al. 2017 [12]	★	★	★	-	-	★★★	Low
Kuroda et al. 2020 [8]	-	★	★	★	★	★★★★	Low
Li et al. 2021 [9]	★	-	★	★	★	★★★★	Low
Xiong et al. 2021 [10]	★	-	★	★	★	★★★★	Low
Zhang et al. 2019 [34]	★	-	★	-	★	★★★	Low

For a detailed description of the criteria used to develop this score, refer to Appendix A. ★ the study is of fair quality in the specific domain

## Data Availability

Raw data extraction will be provided by the first author (A.V.) upon reasonable request.

## Appendix A. Modified Newcastle–Ottawa Scoring Items

(1) *Sample representativeness:*

1 point: Sample size was greater than or equal to 100 participants and exclusion rate was lower than 20%.

0 points: Sample size was fewer than 100 participants or exclusion rate was higher than 20%.

(2) *Sampling technique:*

1 point: Patients recruited consecutively or randomly (randomization criteria clarified).

0 points: Potential convenience sampling or unspecified sampling technique.

(3) *Ascertainment of chronic endometritis diagnosis*:

1 point: The study employed a commonly used histopathological technique (hematoxylin and eosin staining and/or immunohistochemistry with CD-138), with clear diagnostic criteria for chronic endometritis.

0 points: The study employed an infrequently used histopathological technique, without a clear explanation of histological criteria for diagnosis of chronic endometritis.

(4) *Quality of population description:*

1 point: The study reported a clear description of the population (e.g., age, kind of reproductive disorder, diagnostic criteria for the reproductive disorder) with proper measures of dispersion (e.g., mean, standard deviation).

0 points: The study did not report a clear description of the population, incompletely reported descriptive statistics, or did not report measures of dispersion.

(5) *Incomplete outcome data:*

1 point: The study reported complete data about clinical pregnancy rate, ongoing pregnancy/live birth rate, miscarriage rate.

0 points: Selective data reporting cannot be excluded.

The individual components listed above are summed to generate a total modified Newcastle–Ottawa risk of bias score for each study. Total scores range from 0 to 5.

For the total score grouping, studies were judged to be of low risk of bias (≥3 points) or high risk of bias (<3 points).

## Appendix B. General Features of the Studies

**Authors and Year**	**Study Design, Country, and Period of Enrollment**	**Participants and Main Inclusion Criteria**	**IVF-ET Cycle**	**Methods**	**Diagnostic Criteria of CE**	**Groups**	**Outcomes**
Cicinelli et al. 2015 [4]	Retrospective study ------ Italy ------- January 2009–June 2012	106 RIF patients undergoing IVF-ET cycle ------- -Unexplained infertility -Age < 40 years -At least 6 good quality embryos transferred in ≥3 previous IVF/ICSI cycles -Normal karyotype -FSH on day 3 ≤10 mUI/mL -BMI ≤ 30 kg/m^2^ -No previous surgery for myoma and/or endometriosis -No condition interfering with immune system -No antiphospholipid syndrome or thrombophilic condition -No antisperm antibodies	-GnRH-ant with flexible or fixed scheme -rFSH (175–225 IU/day) -U-Hcg (10,000 UI) at follicle size 17 mm (≥2). -Egg retrieval 34 h after ovulation induction -≤3 embryos transferred (of which at least one with good quality) on day 3 of culture -Luteal phase support with vaginal progesterone	-Diagnostic HSC -EB -HIS examination -Endometrial culture -Antibiotic therapy (when appropriate) -Control EB -IVF cycle	1–5 plasma cells/HPF or discrete clusters of <20 plasma cells by CD138 staining	Group A: patients with cured CE (*n* = 46) Group B: patients with persistent CE (*n* = 15)	-Clinical pregnancy rate -Ongoing pregnancy/live birth rate -Miscarriage rate
Demirdag et al. 2021 [11]	Retrospective study ------ Turkey ------- September 2016–December 2019	1164 patients undergoing IVF-ET cycle (232 RIF) ------- -At least 4 good quality embryos transferred in ≥3 previous IVF/ICSI cycles -Age < 40 years -Normal karyotype -Normal uterine cavity -normal antiphospholipid antibody testing -no previous surgery for myoma and/or endometriosis -no male factors infertility - no autoimmune diseases, antiphospholipid antibody syndrome, endocrinological disorders	Exogenous gonadotropins, rFSH alone or with hMG - GnRH antagonist cetrorelix at follicle size ≥14 mm or E2 > 300 pg/mL -rhCG (250 mcg) at follicle size 18 mm (≥2). -Egg retrieval 36 h after ovulation induction -1 to 2 top-quality embryos transferred on day 3 or 5 -Luteal phase support with vaginal progesterone	-EB - HIS examination -Antibiotic therapy (when appropriate) -IVF cycle	≥1 plasma cell/HPF	Group 1: patients with treated CE (*n* = 129) Group 2: patients without CE (*n* = 103) Group 3: patients undergoing the first IVF cycle (*n* = 932)	-Implantation rate -Clinical pregnancy rate -Live birth rate
Fan et al. 2019 [33]	Retrospective study ------ China ------- December 2016–July 2018	141 patients undergoing 1 IVF-ET cycle ------- -At least 2 high quality fresh embryos transferred in a previous IVF/ICSI cycle -Age 20–38 years BMI: 18–25 Kg/m2 - Normal uterine cavity -no endometriosis, adenomyosis, hydrosalpinx, fibroids	-	-EB -HIS examination --IVF cycle	Two methods: ≥1 plasma cell/section or ≥1 plasma cell/mm^2^	Group 1: <1 CD138^+^(*n* = 97) Group 2: ≥1 CD138^+^ (*n* = 44)	-Implantation rate -Clinical pregnancy rate
Hirata et al. 2021 [32]	Prospective study ------ Japan ------- June 2014–September 2017	53 patients undergoing IVF-ET cycle ------- -Age <41 years -Normal uterine cavity -Unexplained infertility - No history of RIF or RPL - No genetic disorders, endocrine diseases or autoimmune diseases	-GnRH-a or GnRH ant protocol -oocyte retrieval and blastocyst freezing -Single blastocyst transfer within 90 days of endometrial tissue sampling with a hormone replacement cycle	-Oocyte retrieval and blastocyst freezing -Diagnostic HSC -EB -HIS examination - single blastocyst transfer	Four different diagnostic criteria: -≥1 plasma cell/10 HPFs -≥2 plasma cell/10 HPFs -≥3 plasma cell/10 HPFs -≥4 plasma cell/10 HPFs	Based on the diagnostic criterion: (≥1; ≥2; ≥3, ≥4) Group A: patients with CE (26; 19; 14; 11) Group B: patients without CE (27; 34; 39; 42)	-Clinical pregnancy rate -Live birth rate -Miscarriage rate
Johnston-MacAnanny et al. 2010 [6]	Prospective study ------ USA ------- 2001–2007	518 RIF patients undergoing IVF-ET cycle 33 with an EB and 485 without an EB ------- -At least 1 good quality embryos transferred in ≥2 previous IVF/ICSI cycles	-GnRH-a or GnRH ant protocol -rFSH alone or with hMG -U-Hcg (5000 or 10000 UI) at follicle size 17 mm (≥2). -Egg retrieval 35 h after ovulation induction -Luteal phase support with vaginal progesterone	-EB -HIS examination -Antibiotic therapy (when appropriate) -Control EB -IVF cycle	≥1 plasma cell/HPF	Group 1: patients with treated CE (*n* = 10) Group 2: patients without CE (*n* = 23) Group 3: RIF patients who did not have an EB (*n* = 485)	-Clinical pregnancy rate -Ongoing pregnancy rate
Kitaya et al. 2017 [12]	Prospective cohort study ------- Japan --- November 2011– July 2014	421 RIF patients undergoing up to three IVF-ET cycle ------- -IVF failure with three or more morphologically good cleavage-stage embryos and/or blastocysts transferred. -No intrauterine pathology	-	-Diagnostic HSC -EB -HIS examination -Endometrial culture -Antibiotic therapy (when appropriate) -Control EB -IVF cycle	ESPDI ≥ 0.25 The endometrial stromal plasmacyte density index (ESPDI) was calculated as the sum of the stromal CD138^+^ cell counts divided by the number of the HPF evaluated.	Group A: patients with cured CE (*n* = 116) Group B: patients with persistent CE (*n* = 4) Group C: patients without CE (*n* = 226)	Clinical pregnancy rate -Ongoing pregnancy/live birth rate -Miscarriage rate
Kuroda et al. 2020 [8]	Cross sectional study ------ Japan ------ June 2018– February 2020	88 infertile women ------ -No intrauterine pathology	-clomiphene citrate or letrozole in combination with rFSH or hMG -hCG 250 μg or nasal buserelin acetate spray 600 μg at follicle size ≥17 mm (≥2) -Egg retrieval 35 h after ovulation induction -Conventional IVF or ICSI - All embryos were cryopreserved at blastocyst developmental stage ≥4 in the Gardner classification using the vitrification method -endometrium prepared for ET via a hormone replacement cycle	-EB -IHC staining -ERA testing -Antiobiotic therapy (when appropriate) -Control EB -IVF cycle	≥5 CD138^+^ plasma cells per 10 random stromal areas at ×400 magnification.	Group A: non CE patients (*n* = 33); Group B: CE patients (*n* = 19) at ERA testing; Group C: cured-CE patients (*n* = 36)	-hCG positive rate -Clinical pregnancy rate -Miscarriage rate -Ongoing pregnancy rate
Li et al. 2021 [9]	Retrospective study ------ China ------ Between 2017 and 2018	716 infertile patients undergoing IVF-ET cycle ------- - <45 years; - endometrial scratching - previous antibiotic treatment for CE	-	- endometrial scratching -EB -HIS examination -IVF	Six different diagnostic criteria - 0 plasma cell/HPF in all of the 30 selected HPFs; -1 plasma cell/hpfs in at least 1 out of 30 selected HPFs; -2 plasma cell/HPFs in at least 1 out of 30 selected HPFs; -3 plasma cell/HPFs in at least 1 out of 30 selected HPFs; -4 plasma cell/HPFs in at least 1 out of 30 selected HPFs; -≥5 plasma cell/HPFs in at least 1 out of 30 selected HPFs;	Group A: 0 CD138^+^/HPF in all of the 30 selected HPFs (*n* = 433); Group B: 1 CD138^+^/HPF in at least 1 out of 30 selected HPFs (*n* = 178); Group C: 2 CD138^+^/HPF in at least 1 out of 30 selected HPFs (*n* = 33); Group D: 3 CD138^+^/HPF in at least 1 out of 30 selected HPFs (*n* = 18); Group E: 4 CD138^+^/HPF in at least 1 out of 30 selected HPFs (*n* = 6); Group F: ≥5 CD138^+^/HPF in at least 1 out of 30 selected HPFs (*n* = 38);	-Clinical pregnancy rate -Live birth rate -Miscarriage rate
Xiong et al. 2021 [10]	Retrospective study ------ China ------ June 2017–June 2018	640 infertile patients undergoing IVF-ET cycle ------- -No antibiotic treatments before the hysteroscopy - age < 40 years; -Normal basal hormone levels (FSH < 10 IU/L and E2 < 60 pg/mL); -BMI < 30 Kg/m^2^; -Normal parental peripheral karyotypes; -Frozen embryo transfer cycles within 6 months after antibiotic treatment - No RPL - no primary ovarian insufficiency - no previous surgery for myoma or endometriosis, - normal uterine cavity	-GnRH a or GnRH ant protocol: -rFSH or hMG -GnRH a or GnRH ant mild stimulation protocol: oral clomiphene citrate 100mg/day + hMG from the fifth day -hCG (10,000 IU) or recombinant hCG (250 mg) when >3 follicles reached a mean diameter of 18 mm; - Oocyte retrieval was performed 36 h after hCG administration; -Luteal phase support with intra- muscular injection of progesterone (60 mg daily) or once daily vaginal progesterone combined with dydrogesterone (10 mg 3 times a day).	-Diagnostic HSC -EB -HIS examination -Antibiotic therapy (when appropriate) -Control EB -IVF cycle	≥1 plasma cell/HPF	Group 1: patients with CD138^+^/HPF = 0 (*n* = 88); Group 2: patients with CD138^+^/HPF 1–4 with antibiotic treatment (*n* = 116); Group 3: patients with CD138^+^/HPF 1–4 without antibiotic treatment (*n* = 199). ------- Group 1: patients with CD138^+^/HPF 0–4 (*n* = 403); Group 2: patients with cured CE (*n* = 211); ------- Group 1: patients with CD138^+^/HPF 0–4 (*n* = 403); Group 2: patients with persistent CE (*n* = 26);	-Implantation rate -Clinical pregnancy rate -Live birth rate -Early pregnancy loss rate -Cumulative live birth rate
Zhang et al. 2019 [34]	Prospective cohort study ------- China ------- February 2015–June 2017	298 RIF patients undergoing 1 IVF-ET cycle ------- -age < 35 years -≥three failed IVF-ET cycles or ≥6 high-quality embryo transferred -Normal uterine cavity -Normal parental peripheral karyotype.	-rFSH (175–225 IU/day) -U-Hcg (10,000 UI) at follicle size 17 mm (≥2) -Egg retrieval 36 h after ovulation induction -≤3 embryos transferred (of which at least one with good quality) on day 3 of culture - Luteal phase support with intramuscular progesterone 60 mg daily	-Diagnostic HSC -EB - HIS examination -intrauterine antibiotic therapy (when appropriate) -Control EB -IVF cycle	≥1 plasma cell/HPF	Group 1: patients without CE (*n* = 126) Group 2: patients with cured CE (*n* = 85) Group 3: patients with persistent CE (*n* = 24)	-Implantation rate -Clinical pregnancy rate -Live birth rate -Clinical loss rate
BMI: body mass index; CE: chronic endometritis; E2: estradiol; EB: endometrial biopsy; ERA testing: endometrial receptivity array testing; ET: embryo transfer; FSH: follicle-stimulating hormone; GnRH-a: GnRH agonist; GnRH-ant: gonadotropin releasing hormone antagonist; HIS: histology; hMG: human menopausal gonadotropin; HPF: high power fields; HSC: hysteroscopy; ICSI: intracytoplasmatic sperm injection; IU: international unit; IVF: in vitro fertilization; RIF: recurrent implantation failure; RPL: recurrent pregnancy loss; rFSH: recombinant FSH; r-Hcg: recombinant human chorionic gonadotropin; U-Hcg: urinary human chorionic gonadotropin.							
